# Characteristics of Websites Presenting Parenteral Supplementation Services in Five European Countries: A Cross-Sectional Study

**DOI:** 10.3390/nu12123614

**Published:** 2020-11-25

**Authors:** Mikołaj Kamiński, Matylda Kręgielska-Narożna, Monika Soczewka, Agnieszka Wesołek, Paulina Rosiejka, Sara Szuman, Paweł Bogdański

**Affiliations:** 1 Department of the Treatment of Obesity and Metabolic Disorders, and of Clinical Dietetics, Poznań University of Medical Sciences, Szamarzewskiego 82/84, 60-569 Poznań, Poland; matylda-kregielska@wp.pl (M.K.-N.); pawelbogdanski73@gmail.com (P.B.); 2 Student Scientific Club of Clinical Dietetics, Department of the Treatment of Obesity and Metabolic Disorders, and of Clinical Dietetics, Poznań University of Medical Sciences, Szamarzewskiego 82/84, 60-569 Poznań, Poland; monika.soczewka2@gmail.com (M.S.); aa.wesolek@gmail.com (A.W.); paulinarosiejka@gmail.com (P.R.); saraszuman@onet.eu (S.S.)

**Keywords:** alternative medicine, dietary supplements, public health, Internet, vitamin

## Abstract

We aimed to characterize the parenteral supplementation services in Czechia, Ireland, Italy, Poland, and the United Kingdom based on their websites. We generated a list of websites by searching Google using the term “vitamin infusion” and selected cities with 250,000 citizens from each analyzed country. All search inputs were performed using the native language. Data on the features of services, indications, contraindications, offered parenteral supplements, and social media activity were obtained. We analyzed 317 websites representing 371 active facilities. Only 6 (1.9%) facilities cited the scientific sources on parenteral supplementation, but these reference were highly biased; 17.4% did not provide information regarding their personnel, while 11.9% indicated the different contraindications. The most common indications were fatigue (62.5%), immunity enhancement (58.0%), anti-aging, and physical activity (51.5%). Approximately, 11.6% of facilities claimed that some parenteral supplements can help manage certain malignancies, while 2.2% claimed that they can help manage fertility problems. The most offered intravenous supplements were vitamins C (57.4%), B12 (47.7%), and B6 (42.3%). The parenteral supplementation market offers numerous ingredients as treatment for general health problems and serious health conditions. Many analyzed websites lacked essential information, which creates concerns for regarding the quality and reliability of the services.

## 1. Introduction

“Vitamin drips” and “vitamin injections” are complementary medicine services consisting of intravenous or subcutaneous administration of vitamins, minerals, or other dietary supplements [1,2]. In this paper, we used the term “parenteral supplementation” [2]. Firstly, most of the ingredients used in the “cocktails” are typical constituents of popular dietary supplements, e.g., minerals, vitamins. Secondly, the mixtures can be provided intravenously as well as subcutaneously. Finally, the companies claim that the intravenous/subcutaneous form of supplementation offers unique advantages over the oral intake [1,3]. The effects of such interventions were poorly investigated; to date, no study has provided high-quality evidence to support the efficacy of parental supplementation [4,5]. At the end of 2019, the NHS medical director warned the public of the potential dangers of these infusions [6]. However, it did not stop companies or celebrities from promoting vitamin intravenous infusions as a panacea [1,7]. Despite the intensive promotion of parenteral supplementation, the phenomenon remains under-researched [2]. The medical, psychological, and legal aspects of commercial infusions are poorly investigated. Most of the reports, also describing fatalities after the administration of supplement drips, were anecdotal and based on journal investigations [8,9]. The scientific reports and recommendations mainly concern the potential efficacy of the intervention using certain substances [10,11] or safety of the drips, in general [12]. Recently, we published the results of a cross-sectional survey study on the experience of Polish individuals using parenteral supplementation services [2]. Most of the respondents used parenteral supplementation to boost their immunity and endurance or deal with fatigue. In most cases, the overall experience with the infusions was described as “good” or “very good”. However, the study group was limited to only 17 individuals.

The analysis of web-derived data provides insights into the under-researched fields of medicine [13]. The most popular source of e-data on health issues are search engine statistics, social media [14], e-forums [15], and websites [16]. The interest of Google users in various dietary supplements has increased in the last years [17]. Several researchers previously investigated the branches of complementary medicine by identifying websites that provide information on alternative treatments [18,19,20,21]. To date, no study focused on companies offering parenteral supplementation services. There is no systematic report on a variety of indications of parenteral supplementation as well as ingredients used in cocktails. We hypothesized that careful analysis of the content of the websites representing facilities offering parenteral supplementation might help understand this branch of complementary medicine. The acquired knowledge may pave the way for further research on this new public health phenomenon.

We aimed to characterize parenteral supplementation services in five European countries based on their websites.

## 2. Materials and Methods

### 2.1. Data Collection

The study was approved by the Bioethical Committee of Poznan University of Medical Sciences (nr of consent 227/20). The study was performed from January to July 2020.

We only included facilities in the cities with at least 250,000 citizens in the Opendatasoft database [22]. The cities with at least 250,000 citizens represent urban centers sizes from L to Global city according to the Organisation for Economic Cooperation and Development and European Commission definitions [23]. Finally, we included 3 cities from Czechia (Brno, Ostrava, and Prague), 1 from Ireland (Dublin), 12 from Italy (Bari, Bologna, Catania, Florence, Bologna, Milan, Naples, Palermo, Rome, Turin, Venice, and Verona), 11 from Poland (Białystok, Bydgoszcz, Gdańsk, Katowice, Kraków, Lublin, Łódź, Poznań, Szczecin, Warsaw, and Wrocław), and 29 from the United Kingdom (Belfast, Birkenhead, Birmingham, Bradford, Bristol, Cardiff, Coventry, Derby, Edinburgh, Glasgow, Islington, Kingston upon Hull, Leeds, Leicester, Liverpool, London, Luton, Manchester, Newport, Nottingham, Plymouth, Preston, Reading, Sheffield, Southend-on-Sea, Stoke-on-Trent, Sunderland, Swansea, and Wolverhampton). These countries were chosen arbitrarily: We wanted to include European countries only, but the research team had limited language skills. To our best knowledge, no study focused on companies offering parenteral services in these countries.

Furthermore, we collected the links to the websites of facilities offering parental supplementation using the Google search engine. Initially, we used Google Ads to compare the search volumes of terms “vitamin infusion,” “vitamin drip,” and “intravenous supplements” in the previous months (July–December 2019). According to Google Ads Keyword Planner, the “vitamin infusion” and its translations in Czech (translated by SS), Italian (translated by SS and PR), and Polish (translated by MK and MS) were the most popular keyword used for searching. In the Google search engine, we typed the term “vitamin infusion” and the city name, in native language, for all analyzed countries. We excluded the links from inactive websites or websites that did not represent facilities offering parenteral supplementation, such as personal websites or blogs. We screened all links generated by Google. The workflow of the links collection is presented in Figure S1.

We prepared a researcher’s form that contained a collection of information from chosen websites. Initially, we used the R-programming language to randomly choose five websites from each country. After analyzing the content of five randomly chosen websites from Poland, three researchers (MK, MS, and MKN) jointly prepared form describing the websites. Furthermore, the form’s initial version was used to collect data from the other five randomly chosen websites representing facilities in Czechia, Ireland, Italy, and the United Kingdom. The other researchers (AW, PR, and SS) independently suggested corrections or new items to the form. The final version of the form is presented in Table S1. The final version of the form was used to subsequently collect the data from all links generated by Google (including websites used previously for the preparation of the initial and final versions of the form). The final form included data on the general characteristics of the facilities (country, link to the website, name of the company, affiliation to the commercial network, address, phone number, and email), social media activity (information on ratings on Google maps, Facebook, Instagram, or Twitter profiles), a feature of the services (experience, information on initial visits, laboratory investigations, location where the service can be performed, and citations of scientific researches), staff, indications and contraindications for parenteral supplementation, offered ingredients, and other services. In this study, the term “indications” referred to the recommendations provided in the websites for parenteral supplementations (both single and multi-ingredient) for different conditions as supportive and/or curative treatment. In the study, we presented other services offered in at least 5% of facilities in at least one country. One website provided information on a chain of facilities. For this reason, we multiplied the record by the number of facilities represented by a website and changed the details of the facilities affiliated to the chains that were different such as address or services offered.

The collection of data from different websites was carried out from January to May 2020. The websites were analyzed by the researchers with excellent/native knowledge of the following languages: Czech (SS), English (AW), Italian (PR), and Polish (MS). All doubts on the characteristics of websites were consulted with MK or MKN.

### 2.2. Data Analysis

All statistical analyses were performed using custom R 3.6.3 code (R Foundation, Vienna, Austria). The data analysis was performed in June–July 2020. The categorical data were presented as number (percentage), while numerical data were expressed as median (interquartile range). We visualized the total number and number of facilities per 100,000 citizens in each included city. Moreover, we computed the total number of facilities per 100,000 citizens in all analyzed cities for each country. Only indications, contraindications, and ingredients that occurred in at least 5% of websites in one country were included in the main results, while the rest is presented in the Supplementary Materials.

## 3. Results

### 3.1. Number of Facilities

**Figure 1:**
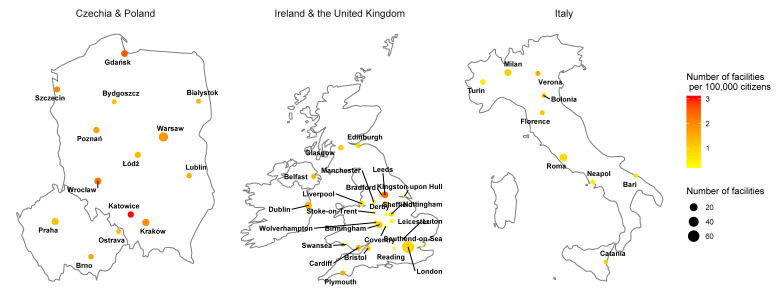
Map of analyzed countries with the number of facilities per 100,000 citizens in each analyzed city.

We included a total of 317 websites, representing 371 facilities. For Italy, we matched 76 webpages of physicians, which mentioned parenteral supplementation as one of the procedures performed by physicians, but these websites were excluded from the study. Most of the included facilities were located in the United Kingdom (n = 154), followed by Poland (n = 121), Italy (n = 58), Czechia (n = 24), and Ireland (n = 14) (Figure 1). The highest number of facilities per 100,000 citizens were observed in Katowice (Poland) (3.06 facilities/100,000 citizens), Gdańsk (Poland) (2.57 facilities/100,000 citizens), and Leeds (the United Kingdom) (2.42 facilities/100,000 citizens). Considering the total population of the included towns, the highest number of facilities was reported in Czechia (1.62 facilities/100,000 citizens), followed by the United Kingdom (1.46 facilities/100,000 citizens), Ireland (1.37 facilities/100,000 citizens), Poland (1.35 facilities/100,000 citizens), and Italy (0.72 facilities/100,000 citizens).

### 3.2. Social Media

The general characteristics and social media activity of the analyzed facilities are presented in Table 1. In Czechia, Ireland, and Italy, more than 20% of facilities were affiliated to a commercial chain; in Poland and the United Kingdom, more than 40% were affiliated to a commercial chain. Overall, parenteral supplementation companies obtained a good consumer rating on Google maps with a median between 4 and 5. The analyzed facilities were active on different social media platforms, mostly in Facebook, followed by Instagram and Twitter.

### 3.3. Features, Staff, and Scientific Information

The features of services and their staff are presented in Table 2. Overall, 6 (1.9%) of all facilities support the use of parenteral supplementation by citing scientific evidence on their websites. In one Polish website, in the section describing offered ingredients, several books related to the use of glutathione [24,25,26] and review on reactive oxygen species were cited [27]. In the second Polish website, in the “Scientific research” section (pl. “Badania naukowe”), the following references were cited: “Publications available on the website: https://www.ncbi.nlm.nih.gov/pubmed and on the website https://www.pum.edu.pl/biblioteka after entering the author’s names” (pl. “Publikacje dostępne na stronie: https://www.ncbi.nlm.nih.gov/pubmed oraz na stronie https://www.pum.edu.pl/biblioteka po wpisaniu nazwisk autora”). However, no article related to parenteral supplementation can be found after typing the names of physicians working in the facility. One facility from Dublin provided links to 22 abstracts from Medline on vitamin C. Among them, there were clinical studies investigating the effects of intravenous vitamin C on the quality of life of individuals with malignancy [28,29], cancer markers [30,31], sepsis [32,33], and C-reactive protein level in hemodialysis patients [34]. Most of the other articles were reviews or basic science research. Other websites mostly cited reviews on offered micronutrients and meta-analyses on the effects of zinc lozenges on common cold [35]. In two websites from the United Kingdom, we found a reference regarding “Myers’ cocktail” [36]. In one case, the scientific section was inaccessible during the preparation of our manuscript (July 2020), and no citation was found. The substantial number (17.4%) of companies did not provide information regarding their personnel.

### 3.4. Indications and Contraindications

The most popular indications were fatigue (62.5%), immunity enhancement (58.0%), anti-aging, and physical activity (51.5%) (Table 3). Less than one of the eight companies provided information on contraindications to the procedure; among them, the most commonly described were hypersensitivity, pregnancy, and renal insufficiency (Table 3).

### 3.5. Type of Supplementation

Many facilities offered multi-ingredient intravenous supplementation, but a substantial number did not provide the exact information on the ingredients of cocktails (Table 4). Among intravenously administered supplements, the most commonly offered were vitamin C (57.4%), vitamin B12 (47.7%), and vitamin B6 (42.3%) (Table 4). The subcutaneous supplementation was offered mainly in Ireland and the United Kingdom.

The indications, contraindications, and ingredients that were not described in at least 5% of websites in each country are presented in Table S2. Parenteral supplementation was recommended as treatment for fertility problems in five facilities in Poland and in three facilities in the United Kingdom (Table S2). The other services offered in the analyzed facilities are presented in Table S3. Interestingly, the parenteral supplementation and beauty salon services were commonly provided by the same companies.

## 4. Discussion

We analyzed the websites representing facilities offering parenteral supplementation in five European countries. We found that many websites lack essential information on parenteral supplementation. Simultaneously, the vitamin infusions were offered as treatment for both general ailments and serious conditions.

### 4.1. Main Findings

This study was the first to describe the content of websites of facilities offering parenteral supplementation. Overall, vitamin intravenous infusions were the most popular parenteral supplement offered in Czechia and the United Kingdom. The high number of facilities per 100,000 citizens in cities like Katowice, Gdańsk, and Leeds may be due to the high number of citizens living in surrounding towns.

The facilities offering parenteral supplementation are active on social media, with audiences between several hundred and several dozen thousands. Social media is a popular channel to promote alternative medicine methods and might be a valuable source for research [37,38,39]. Interestingly, the reviews on Google maps profile were generally positive, which may suggest that consumers had a positive experience with the facilities. However, personal belief and subjective response to vitamin infusions require further research [2]. The social media profiles of the facilities offering parenteral supplementation might be a good platform to reach the consumers.

Most of the facilities did not indicate their years of experience; thus, many companies might have a history far shorter than the median of 9 years. However, a substantial number of services are offered over a decade, which means that vitamin infusions is not a novel method; rather, it is an old method that gained attention just recently. Only Polish facilities had a median experience of less than 5 years, which suggests that the branch recently developed in this country.

Parenteral supplementation had no solid scientific evidence supporting its advertised beneficial properties [4,5]. A minority of the companies defend their standby citing scientific researches. Most of the references cited popular science books or non-systematic reviews. However, these references lacked the use of a placebo group as a comparator or did not indicate strong clinical outcomes. Interestingly, one webpage cited articles regarding the feasibility of intravenous vitamin C for sepsis—a condition that cannot be treated in ambulatory settings such as vitamin infusion facilities. None of the articles reported parenteral supplementation for common indications such as fatigue, physical endurance, or immunity enhancement.

In this study, the following two features were assessed in all facilities as qualifications for parenteral supplementation: description of initial visits and the opportunity to perform lab investigations. However, most of the analyzed facilities lacked complete information on both aspects. Because most of the ingredients were vitamins, macronutrients, or micronutrients, the status of these chemical compounds in the blood should be assessed before deciding to receive the infusion. Similarly, the initial visit is crucial to determine whether a consumer has a contraindication to the parenteral supplementation. The parenteral administration has universal contraindications, such as known hypersensitivity to any ingredient. The poorly investigated agents or the agents that are not commonly used among pregnant or breastfeeding women should be contraindicated in this group. Moreover, decompensated heart failure or end-stage chronic kidney is a contraindication to obtain excessive intravenous fluid. There are many other specific contraindications, but those mentioned above are the general contraindications. All consumers should be informed regarding them. Another concern is related to the missing information on medical professionals who work in the facilities, and who perform infusions and/or injections. Many of the companies claimed to employ dieticians, psychologists, physiotherapists, or cosmetologists, which may be associated with other offered services. However, in the analyzed countries, only physicians, nurses/midwives, or paramedics have the right to administer agents parenterally. Hence, the appropriate personnel who is certified to administer the vitamin infusions if no information is provided on the website remains unclear. Taken together, a lack of essential information creates concerns regarding the safety and reliability of the services.

Alternative treatments are often offered as management for conditions with highly subjective symptoms [40]. Indeed, most of the recommended indications were general health problems. In such settings, the placebo effect tends to be more perceptible [40]. The promotion via social media and positive comments or good ratings may support the enhanced placebo effects through social proof phenomenon [41]. The consumers may perceive that many others were satisfied by the service. For these reasons, parenteral supplement consumers may experience health improvements. Positive attitude and the desire to improve health are essential even for individuals with malignancy [42]. However, whether personal funds should be spent on an evidence-based intervention that is not gaining the placebo effect due to the use of vitamin intravenous infusions needs to be explored further. Another concern is the number of consumers that discontinued conventional treatment as they were persuaded by alternative medicine enthusiasts who shared their positive experience after receiving the supplements.

The most offered ingredients, such as vitamins, macronutrients or micronutrients, glutathione, and amino acids, are dietary supplements and are among the most popular searches in Google [17]. Many of the searched ingredients are used in hospital settings such as electrolytes, vitamin B complex, or glucose. Probably, many of the intravenous formulas used in facilities providing parenteral supplementation were purchased from companies that produce them for clinical practice. Performing a detailed analysis of the ingredients allowed in each country is beyond the scope of this study. However, the very concern is offering solutions that are not commonly used by clinicians. Further studies should focus on the source of parenteral supplements that are not also used in conventional medicine.

In this cross-sectional study, we characterized the companies offering parenteral supplementation based on their websites. This branch of complementary medicine seemed to be a wellness service rather than an evidence-based intervention. Although previous studies on parenteral supplementation were at high risk of bias, the absence of evidence does not mean that such practices were not utilized. The freedom of action on the market is an essential foundation of Western Civilization. However, this is an unjustifiable and unfair competition with conventional medicine by claiming the unproven effects of the supplementation. Moreover, many websites lack crucial information, which creates concerns about the quality and reliability of the services. The efficacy and safety of vitamin infusions services require further research.

### 4.2. Limitations

Our study has several limitations. We created an original data collection form from the initial analysis of a sample of 5 websites from each country. The relatively small sample of initially analyzed websites could be a source of bias in constructing the form. The analyzed countries were chosen arbitrarily, and the results cannot be extrapolated to the other countries. The arbitral choice resulted in including Ireland, which had only one city with at least 250,000 citizens, and a diverse number of found websites, e.g., 121 in Poland and only 14 in Ireland. For this reason, the characteristics of the services in these countries are not comparable. We included services offered in the cities with more than 250,000 citizens; thus, the analysis does not include services provided in smaller towns and does not represent the whole picture of each country’s parenteral supplementation industry. The study only focuses on facilities that possess websites. We cannot assess the number of facilities that do not have a website and the representativeness of our sample. Furthermore, the information on services is not definitive, and consumers may obtain different information or ingredients in the facility. Finally, the study is cross-sectional, and the content of the analyzed websites may be outdated during the period data collection or may change in the future.

## 5. Conclusions

The market of parenteral supplementation offers numerous ingredients as treatment for general health problems and serious health conditions. Many analyzed websites lack essential information, which creates concerns regarding the quality and reliability of the services.

## Figures and Tables

**Table 1:** General information of facilities offering parenteral supplementation, and their social media activity. Data presented as number (%)/median (interquartile range).

Variables	All n = 317	Czechia n = 24	Ireland n = 14	Italy n = 58	Poland n = 121	United Kingdom n = 154
Facilities in commercial chain (n)	144 (38.8%)	7 (29.2%)	3 (21.4%)	13 (22.4%)	54 (44.6%)	67 (43.5%)
Information on address (n)	345 (93.0%)	24.00 (100.0%)	14 (100.0%)	58 (100.0%)	103 (85.1%)	146 (94.8%)
Information on e-mail address (n)	335 (90.3%)	21.00 (87.5%)	13 (92.9%)	54 (93.1%)	121 (100.0%)	141 (91.6%)
Information on phone number (n)	369 (99.5%)	24.00 (100.0%)	13 (92.9%)	58 (100.0%)	106 (87.6%)	153 (99.4%)
**Social Media**						
Review in Google Maps (n)	283 (76.3%)	16 (66.7%)	10 (71.4%)	51 (87.9%)	83 (68.6%)	123 (79.9%)
Rating in Google Maps (n)	5.0 (5.0–5.0)	4.0 (3.5–4.4)	5.0 (4.7–5.0)	4.1 (4.5–5.0)	4.6 (4.3–5.0)	4.7 (4.5–5.0)
Number of Google ratings per facility (n)	20 (8–48)	19 (7–27)	8 (24–119)	12 (6–32)	19 (8–46)	28 (10–100)
Number of Facebook pages (n)	313 (84.4%)	12 (50.0%)	12 (85.7%)	55 (94.8%)	97 (80.1%)	137 (89.0%)
Facebook likes (n)	1149 (4295–2846)	1607 (762–31,500)	2071 (309–5488)	1527 (763–3776)	1000 (332–1963)	1070 (477–3322)
Facebook followers (n)	1249 (438–2906)	1634 (783–31,170)	2081 (315–5470)	1539 (782–3804)	1042 (337–1995)	1101 (514–3363)
Instagram profiles (n)	229 (61.7%)	7 (29.2%)	10 (71.4%)	30 (51.7%)	61 (50.4%)	121 (78.6%)
Instagram followers (n)	1289 (402–5460)	12,325 (1293–20,000)	781 (245–9570)	783 (402–5081)	536 (97–1048)	2542 (754–9715)
Instagram posts (n)	261 (70–542)	561 (78–864)	253 (49–498)	179 (63–461)	74 (23–180)	436 (213–629)
Twitter pages (n)	139 (37.5%)	2 (8.3%)	8 (57.1%)	9 (15.5%)	4 (3.3%)	113 (73.4%)
Twitter followers (n)	430 (80–1663)	87 (47–127)	177 (76–762)	14 (9–69)	19 (0–1974)	81 (146–1386)

**Table 2:** Features of the services offering parenteral supplementation and their staff. Data presented as number (%)/median (interquartile range).

Variables	All n = 317	Czechia n = 24	Ireland n = 14	Italy n = 58	Poland n = 121	United Kingdom n = 154
**Feature of the Services**						
Information on years of experience (n)	142 (38.3%)	9 (37.5%)	8 (57.1%)	13 (22.4%)	59 (48.8%)	53 (34.4%)
Experience (years)	9.0 (3.0–15.0)	9.0 (9.0–9.0)	11.5 (9.0–16.3)	18.0 (10.0–20.0)	4.0 (2.0–13.5)	10.0 (5.0–14.0)
Initial visit (n)	165 (44.5%)	11 (45.8%)	6 (42.9%)	9 (15.5%)	54 (44.6%)	85 (55.2%)
Lab investigations (n)	82 (22.1%)	6 (25.0%)	4 (28.6%)	9 (15.5%)	33 (27.3%)	30 (19.5%)
Service in own premise (n)	341 (91.9%)	24 (100.0%)	14 (100.0%)	58 (100.0%)	96 (79.3%)	149 (96.8%)
Services at the place indicated by the customer (n)	53 (14.3%)	1 (4.2%)	0 (0.0%)	3 (5.2%)	35 (28.9%)	14 (9.1%)
Citation of scientific evidence on websites (n)	6 (1.9%)	2 (8.3%)	1 (7.1%)	0 (0.0%)	2 (1.7%)	3 (2.0%)
**Staff**						
Information on personnel (n)	310 (83.6%)	24 (100.0%)	11 (78.6%)	58 (100.0%)	81 (66.9%)	136 (88.3%)
Physician (n)	248 (66.9%)	24 (100.0%)	8 (57.14%)	54 (93.1%)	72 (59.5%)	90 (58.4%)
Nurse and/or midwife (n)	74 (19.95%)	4 (16.7%)	1 (7.1%)	7 (12.1%)	25 (20.7%)	37 (24.0%)
Dietician (n)	45 (12.13%)	0 (0.0%)	0 (0.0%)	17 (29.3%)	18 (14.9%)	10 (6.5%)
Cosmetologist (n)	32 (8.63%)	0 (0.0%)	2 (14.3%)	5 (8.6%)	9 (7.4%)	16 (10.4%)
Psychologist (n)	11 (2.96%)	0 (0.0%)	0 (0.0%)	10 (17.2%)	1 (0.8%)	0 (0.0%)
Paramedic (n)	8 (2.16%)	0 (0.0%)	0 (0.0%)	2 (3.5%)	5 (4.1%)	1 (0.7%)
Physiotherapist (n)	7 (1.89%)	0 (0.0%)	0 (0.0%)	5 (8.6%)	2 (1.7%)	0 (0.0%)

**Table 3:** Indications and contraindications for parenteral supplementation. Data presented as number (%).

Variables	All n = 317	Czechia n = 24	Ireland n = 14	Italy n = 58	Poland n = 121	United Kingdom n = 154
**Indications**						
Vitamin and/or micronutrients deficiency (n)	186 (50.1%)	18 (75.0%)	2 (14.3%)	1 (1.7%)	98 (81.0%)	67 (43.5%)
Physically active individuals (n)	191 (51.5%)	11 (45.8%)	3 (21.4%)	3 (5.2%)	92 (76.0%)	82 (53.3%)
Fatigue (n)	232 (62.5%)	20 (83.3%)	6 (42.9%)	4 (6.9%)	89 (73.6%)	113 (73.4%)
Excessive stress (n)	140 (37.7%)	19 (79.2%)	3 (21.4%)	5 (8.6%)	69 (57.0%)	44 (28.6%)
Concentration loss or need of mental boost (n)	47 (12.7%)	1 (4.2%)	0 (0.0%)	1 (1.7%)	29 (24.0%)	15 (9.7%)
Symptoms of depressive disorders (n)	81 (21.8%)	14 (58.3%)	0 (0.0%)	1 (1.7%)	33 (27.3%)	33 (21.4%)
Hangover (n)	124 (33.4%)	1 (4.2%)	2 (14.4%)	4 (6.9%)	83 (68.6%)	34 (22.1%)
Immunity enhancement (n)	215 (58.0%)	23 (95.8%)	5 (35.7%)	2 (3.5%)	79 (65.3%)	106 (68.8%)
Dehydration (n)	108 (29.1%)	1 (4.2%)	5 (35.7%)	2 (3.5%)	50 (41.3%)	50 (32.5%)
Detoxification (n)	153 (41.2%)	12 (50.0%)	3 (21.4%)	5 (8.6%)	48 (39.7%)	85 (55.2%)
Anti-aging (n)	191 (51.5%)	10 (41.7%)	4 (28.6%)	48 (82.8%)	38 (31.4%)	91 (59.1%)
Beauty improvement (n)	190 (51.2%)	0 (0.0%)	10 (71.4%)	54 (93.1%)	30 (24.9%)	96 (62.3%)
Weight loss (n)	87 (23.5%)	5 (20.8%)	2 (14.3%)	1 (1.7%)	28 (23.1%)	51 (33.1%)
Improvement of libido (n)	41 (11.1%)	4 (16.7%)	0 (0.0%)	0 (0.0%)	31 (25.6%)	6 (3.9%)
For specific diseases (n)	77 (20.8%)	19 (79.2%)	3 (21.4%)	0 (0.0%)	32 (26.5%)	23 (14.9%)
Malignancy (n)	43 (11.6%)	16 (66.7%)	1 (7.1%)	0 (0.0%)	19 (15.7%)	7 (4.6%)
**Contraindications**						
Information on contraindications (n)	44 (11.9%)	5 (20.8%)	0 (0.0%)	4 (6.9%)	22 (18.2%)	13 (8.4%)
Hypersensitiveness (n)	19 (5.1%)	1 (4.2%)	0 (0.0%)	2 (3.5%)	10 (8.3%)	6 (3.9%)
Pregnancy (n)	17 (4.6%)	1 (4.2%)	0 (0.0%)	2 (3.5%)	11 (9.2%)	3 (1.95%)
Renal insufficiency (n)	15 (4.0%)	2 (8.3%)	0 (0.0%)	0 (0.0%)	7 (5.8%)	6 (3.9%)
Certain drugs (n)	8 (2.2%)	1 (4.2%)	0 (0.0%)	0 (0.0%)	7 (5.8%)	0 (0.0%)
Hemochromatosis (n)	8 (2.2%)	3 (12.5%)	0 (0.0%)	0 (0.0%)	3 (2.5%)	2 (1.3%)
Renal stones (n)	5 (1.4%)	4 (16.7%)	0 (0.0%)	0 (0.0%)	2 (1.7%)	0 (0.0%)

**Table 4:** Parenteral supplementation ingredients. Data presented as number (%).

Variables	All n = 317	Czechia n = 24	Ireland n = 14	Italy n = 58	Poland n = 121	United Kingdom n = 154
Multi-ingredient infusion (Cocktails) (n)	248 (66.9%)	8 (33.3%)	6 (42.9%)	7 (12.1%)	114 (94.2%)	113 (73.4%)
No information on exact cocktails ingredients (n)	86 (23.2%)	5 (20.8%)	1 (7.1%)	5 (8.62%)	57 (47.1%)	18 (11.7%)
**Electrolytes, Macro-/Micronutrients**						
Magnesium (n)	151 (40.7%)	4 (16.7%)	6 (42.9%)	3 (5.2%)	56 (46.3%)	82 (53.3%)
Zinc (n)	102 (27.5%)	0 (0.0%)	3 (21.4%)	2 (3.5%)	33 (27.3%)	64 (41.6%)
Selenium (n)	92 (24.8%)	0 (0.0%)	1 (7.1%)	2 (3.5%)	33 (27.3%)	56 (36.4%)
Multi-electrolyte fluid (n)	66 (17.8%)	2 (8.3%)	2 (14.3%)	1 (1.7%)	34 (28.1%)	27 (17.5%)
Calcium (n)	66 (17.8%)	1 (4.2%)	3 (21.4%)	0 (0.0%)	14 (11.6%)	48 (31.2%)
NaCl 0,9% (n)	44 (11.9%)	1 (4.2%)	3 (21.4%)	1 (1.7%)	11 (9.1%)	28 (18.2%)
Iron (n)	43 (11.6%)	0 (0.0%)	1 (7.1%)	0 (0.0%)	19 (15.7%)	23 (14.9%)
Chromium (n)	32 (8.6%)	0 (0.0%)	0 (0.0%)	2 (3.5%)	22 (18.2%)	8 (5.2%)
Iodine (n)	30 (8.1%)	0 (0.0%)	0 (0.0%)	0 (0.0%)	24 (19.8%)	6 (3.9%)
Molybdenum (n)	22 (5.9%)	0 (0.0%)	0 (0.0%)	0 (0.0%)	20 (16.5%)	2 (1.3%)
Copper (n)	20 (5.4%)	0 (0.0%)	0 (0.0%)	0 (0.0%)	16 (13.2%)	4 (2.6%)
Mangan (n)	14 (3.8%)	0 (0.0%)	0 (0.0%)	0 (0.0%)	11 (9.1%)	3 (2.0%)
Fluor (n)	7 (1.9%)	0 (0.0%)	0 (0.0%)	0 (0.0%)	6 (5.0%)	1 (0.7%)
**Vitamins**						
Vitamin A (n)	54 (14.6%)	1 (4.2%)	0 (0.0%)	0 (0.0%)	46 (38.0%)	7 (4.6%)
Vitamin B1 (n)	149 (40.2%)	3 (12.5%)	6 (42.9%)	0 (0.0%)	52 (43.0%)	88 (57.1%)
Vitamin B2 (n)	147 (39.6%)	3 (12.5%)	6 (42.9%)	0 (0.0%)	51 (42.2%)	87 (56.5%)
Vitamin B3/PP (n)	138 (37.2%)	3 (12.5%)	6 (42.9%)	0 (0.0%)	46 (38.0%)	83 (53.9%)
Vitamin B5 (n)	155 (41.8%)	3 (12.5%)	6 (42.9%)	1 (1.7%)	52 (43.0%)	93 (60.4%)
Vitamin B6 (n)	157 (42.3%)	5 (20.8%)	6 (42.9%)	0 (0.0%)	52 (43.0%)	94 (61.0%)
Vitamin B7 / H (n)	141 (38.0%)	3 (12.5%)	6 (42.9%)	1 (1.7%)	52 (43.0%)	79 (51.3%)
Vitamin B9 / folic acid (n)	141 (38.0%)	3 (12.5%)	6 (42.9%)	0 (0.0%)	51 (42.2%)	81 (52.6%)
Vitamin B12 (n)	177 (47.7%)	5 (20.8%)	6 (42.9%)	2 (3.5%)	60 (49.6%)	104 (67.5%)
Vitamin C (n)	213 (57.4%)	22 (91.7%)	5 (35.7%)	5 (8.6%)	83 (68.6%)	98 (63.6%)
Vitamin D3 (n)	74 (20.0%)	2 (8.3%)	1 (7.1%)	0 (0.0%)	44 (36.4%)	27 (17.5%)
Vitamin E (n)	51 (13.8%)	1 (4.2%)	1 (7.1%)	0 (0.0%)	31 (25.6%)	18 (11.7%)
Vitamin K (n)	30 (8.1%)	0 (0.0%)	1 (7.1%)	0 (0.0%)	18 (14.9%)	11 (7.1%)
**Other Supplements**						
Glutathione (n)	127 (34.2%)	4 (16.7%)	5 (35.7%)	3 (5.2%)	34 (28.1%)	81 (52.6%)
Amino acids (n)	115 (31.0%)	0 (0.0%)	4 (28.6%)	2 (3.5%)	21 (17.4%)	88 (57.1%)
Alphalipoic acid (n)	52 (14.0%)	1 (4.2%)	2 (14.3%)	0 (0.0%)	37 (30.6%)	12 (7.8%)
Coenzyme Q10 (n)	39 (10.5%)	0 (0.0%)	1 (7.1%)	1 (1.7%)	17 (14.1%)	20 (13.0%)
Glucose (n)	34 (9.2%)	1 (4.2%)	1 (7.1%)	1 (1.7%)	26 (21.5%)	5 (3.3%)
Solcoseryl (n)	34 (9.2%)	0 (0.0%)	1 (7.1%)	0 (0.0%)	29 (24.0%)	4 (2.6%)
Choline (n)	12 (3.2%)	2 (8.3%)	0 (0.0%)	0 (0.0%)	5 (4.1%)	5 (3.3%)
Collagen (n)	11 (3.0%)	0 (0.0%)	0 (0.0%)	0 (0.0%)	2 (1.7%)	9 (5.8%)
Acetylcysteine (n)	9 (2.4%)	1 (4.2%)	0 (0.0%)	0 (0.0%)	8 (6.6%)	0 (0.0%)
Medications (n)	9 (2.4%)	3 (12.5%)	1 (7.1%)	0 (0.0%)	3 (2.5%)	2 (1.3%)
Ginkgo biloba (n)	5 (1.4%)	0 (0.0%)	1 (7.1%)	0 (0.0%)	1 (0.8%)	3 (2.0%)
**Types of Subcutaneous Supplements**						
Vitamin A (n)	17 (4.6%)	0 (0.0%)	7 (50.0%)	4 (6.9%)	0 (0%)	8 (5.2%)
Vitamin C (n)	42 (11.3%)	0 (0.0%)	5 (35.7%)	4 (6.9%)	0 (0%)	31 (20.1%)

## Supplementary Materials

The following are available online at https://www.mdpi.com/2072-6643/12/12/3614/s1. Figure S1: The workflow of the collection process of links to the websites. The first step was to choose the city (the most left upper box) to add it to the query (the most right upper box). We checked all websites suggested by Google and saved the links of websites representing parenteral supplementation services. Cz—Czech, En—English, It—Italian, Pl—Polish. Table S1: Form for collecting characteristics from websites of parental supplementation services. Table S2: Indications, contraindications, and ingredients mentioned in less than in 5% of websites in each country. Data presented as number (%). Table S3: The other services offered in the analyzed facilities.

## References

[1] Rimmer A. (2019). Sixty seconds on … vitamin drips. BMJ.

[2] Kamiński M., Kręgielska-Narożna M., Soczewka M., Bogdański P. (2020). Why Polish patients use vitamin drips services: Results of a preliminary cross-sectional survey. Pol. Arch. Intern. Med..

[3] Sams L. (2019). Science or not, IV “wellness” drips are booming. Financial Review.

[4] Bilg R. (2017). A Rapid Evidence Assessment on the Effectiveness of Intravenous Mega-Dose Multivitamins on Fibromyalgia, Chronic Fatigue, Cancer, and Asthma. Master’s Thesis.

[5] Gavura S. A Closer Look at Vitamin Injections. Science Based Medicine Website. https://sciencebasedmedicine.org/a-closer-look-at-vitamin--injections.

[6] (2019). England’s Top Doctor Slams ‘Exploitative’ Party Drips.

[7] Iqbal N. (2019). Celebrities help the £500 vitamin jab go mainstream. The Guardian.

[8] Vitamin Drips. Hit or Great Scam? [In Polish] Dzień Dobry TVN Website. https://dziendobry.tvn.pl/a/wlewy-witaminowe-hit-czy-wielkie-oszustwo.

[9] Death of a 36-Year Old Woman in a Poznań Hospital. She Came from a Natural Medicine Clinic [In Polish]. https://www.wprost.pl/polityka/10185708/smierc-36-latki-w-poznanskim-szpitalu-trafila-tam-z-kliniki-medycyny-naturalnej.html.

[10] (2019). Unsafe Use of Glutathione as Skin Lightening Agent.

[11] Madsen B.K., Hilscher M., Zetner D., Rosenberg J. (2019). Adverse reactions of dimethyl sulfoxide in humans: A systematic review. F1000Research.

[12] Lenz J.R., Degnan D., Hertig J.B., Stevenson J.G. (2017). A Review of Best Practices for Intravenous Push Medication Administration. J. Infus. Nurs..

[13] Eysenbach G. (2011). Infodemiology and Infoveillance. Am. J. Prev. Med..

[14] Sinnenberg L., Buttenheim A.M., Padrez K., Mancheno C., Ungar L., Merchant R.M. (2017). Twitter as a Tool for Health Research: A Systematic Review. Am. J. Public Health.

[15] Kamiński M., Borger M., Prymas P., Muth A., Stachowski A., Łoniewski I., Marlicz W. (2020). Analysis of Answers to Queries among Anonymous Users with Gastroenterological Problems on an Internet Forum. Int. J. Environ. Res. Public Health.

[16] Baudischova L., Zubrova J., Pokladnikova J., Jahodar L. (2018). The quality of information on the internet relating to top-selling dietary supplements in the Czech Republic. Int. J. Clin. Pharm..

[17] Kamiński M., Kręgielska-Narożna M., Bogdanski P. (2020). Determination of the Popularity of Dietary Supplements Using Google Search Rankings. Nutrients.

[18] Page S.A., Mannion C., Bell L.H., Verhoef M. (2010). CAM information online: An audit of Internet information on the “Bill Henderson Protocol”. Complement. Ther. Med..

[19] Ernst E., Schmidt K. (2002). ‘Alternative’ cancer cures via the Internet?. Br. J. Cancer.

[20] Jensen R.K., Agersted M.E.I., Nielsen H.A., O’Neill S. (2020). A cross-sectional study of website claims related to diagnoses and treatment of non-musculoskeletal conditions. Chiropr. Man. Ther..

[21] Sharma V., Holmes J.H., Sarkar I.N. (2016). Identifying Complementary and Alternative Medicine Usage Information from Internet Resources: A Systematic Review. Methods Inf. Med..

[22] Opendatasoft. Geonames—All Cities with a Population. https://data.opendatasoft.com/explore/dataset/geonames-all-cities-with-a-population-1000%40public/table/?disjunctive.country.

[23] Dijkstra L., Poelman H. (2012). Cities in Europe. The New OECD-EC Definition.

[24] Witek B., Tabara J., Świderska-Kołacz G., Kołątaj A. (2015). The Reduced Glutathione Level and Glutathione Enzymes Activity in Connection with the Blood Storage.

[25] Labrou N., Labrou N., Flemetakis E. (2013). Glutathione: Biochemistry, Mechanisms of Action & Biotechnological Implications.

[26] Gutman J., Schettini S., Bounous G. (2008). Glutathione: Your Key to Health.

[27] Ługowski M., Saczko J., Kulbacka J., Banaś T. (2011). Reactive oxygen and nitrogen species. Pol. Merkur. Lekarski.

[28] Vollbracht C., Schneider B., Leendert V., Weiss G., Auerbach L., Beuth M.J. (2011). Intravenous vitamin C administration improves quality of life in breast cancer patients during chemo-/radiotherapy and aftercare: Results of a retrospective, multicentre, epidemiological cohort study in Germany. In Vivo.

[29] Yeom C.H., Jung G.C., Song K.J. (2007). Changes of Terminal Cancer Patients’ Health-related Quality of Life after High Dose Vitamin C Administration. J. Korean Med Sci..

[30] Mikirova N.A., Casciari J., Rogers A., Taylor P. (2012). Effect of high-dose intravenous vitamin C on inflammation in cancer patients. J. Transl. Med..

[31] Mikirova N.A., Casciari J., Riordan N.H., Hunninghake R. (2013). Clinical experience with intravenous administration of ascorbic acid: Achievable levels in blood for different states of inflammation and disease in cancer patients. J. Transl. Med..

[32] Fowler A.A., A Syed A., Knowlson S., Sculthorpe R., Farthing D., Dewilde C., A Farthing C., Larus T.L., Martin E., Brophy D.F. (2014). Phase I safety trial of intravenous ascorbic acid in patients with severe sepsis. J. Transl. Med..

[33] Marik P.E., Khangoora V., Rivera R., Hooper M.H., Catravas J. (2017). Hydrocortisone, Vitamin C, and Thiamine for the Treatment of Severe Sepsis and Septic Shock. Chest.

[34] Biniaz V., Shermeh M.S., Ebadi A., Tayebi A., Einollahi B. (2013). Effect of Vitamin C Supplementation on C-reactive Protein Levels in Patients Undergoing Hemodialysis: A Randomized, Double Blind, Placebo-Controlled Study. Nephro-Urol. Mon..

[35] Hemilä H. (2017). Zinc lozenges and the common cold: A meta-analysis comparing zinc acetate and zinc gluconate, and the role of zinc dosage. JRSM Open.

[36] Gaby A.R. (2002). Intravenous nutrient therapy: The “Myers’ cocktail”. Altern. Med. Rev. J. Clin. Ther..

[37] Delgado-López P.D., Corrales-García E.M. (2018). Influence of Internet and Social Media in the Promotion of Alternative Oncology, Cancer Quackery, and the Predatory Publishing Phenomenon. Cureus.

[38] Hansen M.M., Grajales F.J., Martin-Sanchez F., Bamidis P.D., Miron-Shatz T. (2013). Social Media for the Promotion of Holistic Self-Participatory Care: An Evidence Based Approach. Yearb. Med. Inform..

[39] George D.R., Rovniak L.S., Kraschnewski J.L. (2013). Dangers and Opportunities for Social Media in Medicine. Clin. Obstet. Gynecol..

[40] Kaptchuk T.J. (2002). The Placebo Effect in Alternative Medicine: Can the Performance of a Healing Ritual Have Clinical Significance?. Ann. Intern. Med..

[41] Cialdini R.B. (1993). Influence: The Psychology of Persuasion.

[42] Carver C.S., Smith R.G., Antoni M.H., Petronis V.M., Weiss S., DerHagopian R.P. (2005). Optimistic Personality and Psychosocial Well-Being During Treatment Predict Psychosocial Well-Being Among Long-Term Survivors of Breast Cancer. Health Psychol..

