# Supplementary figures and images for: Characteristics of Websites Presenting Parenteral Supplementation Services in Five European Countries: A Cross-Sectional Study
Source: Nutrients. 2020 Nov 25;12(12):3614. doi: 10.3390/nu12123614 (PMC7760744; doi:10.3390/nu12123614)

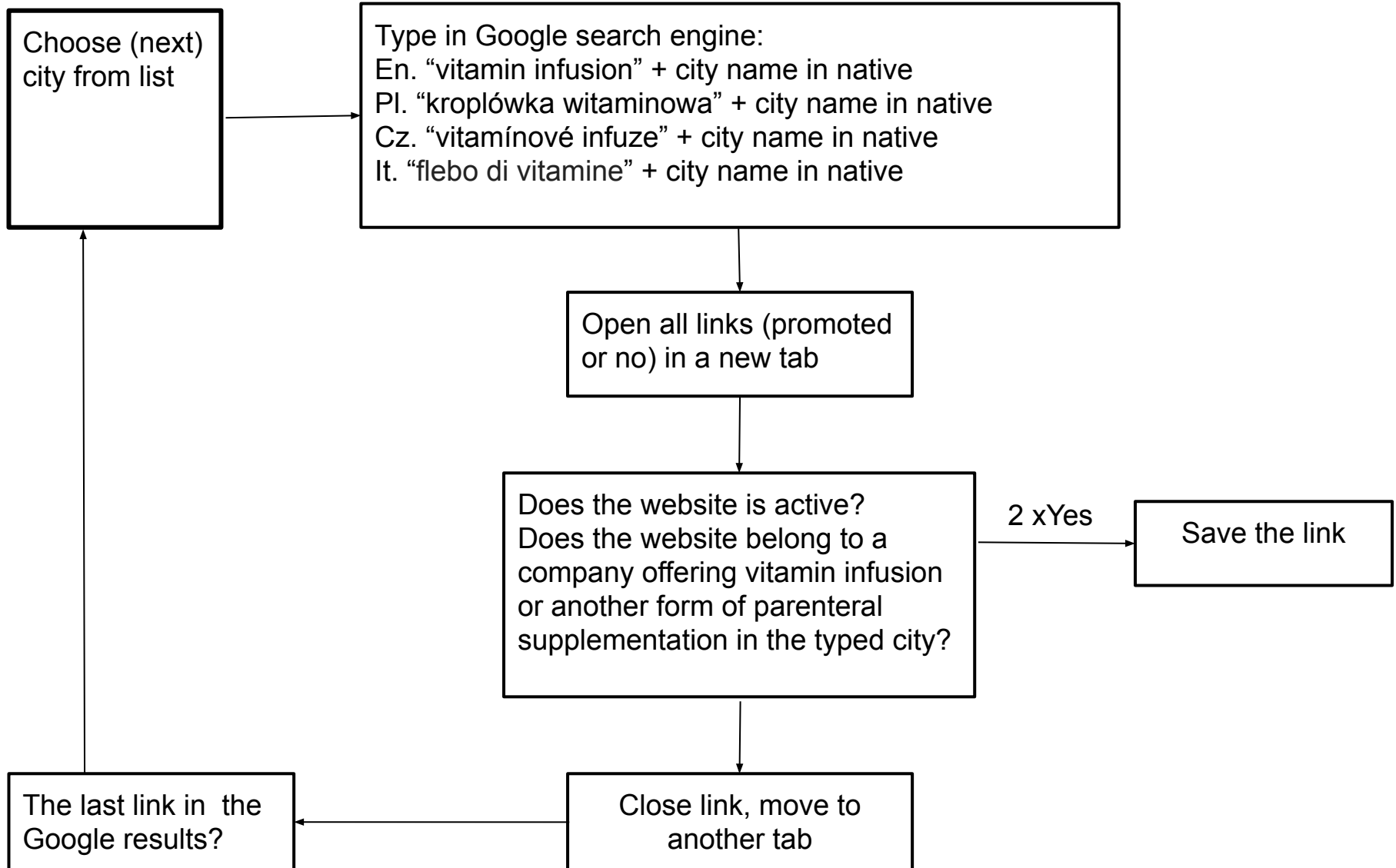

Supplement: Supplementary file 1 [file nutrients-12-03614-s001.zip › Figure S1.pdf]
