# Supplementary material for: Characteristics of Websites Presenting Parenteral Supplementation Services in Five European Countries: A Cross-Sectional Study
Source: Nutrients. 2020 Nov 25;12(12):3614. doi: 10.3390/nu12123614 (PMC7760744; doi:10.3390/nu12123614)
Supplement: Supplementary file 1 [file nutrients-12-03614-s001.zip › Table S1.doc]

Table S1

Form for collecting characteristics from websites of parental supplementation services

A)

| **Name of variable** | **Data format** |
| --- | --- |
| **General information** | |
| Country | text |
| Date of collection | date |
| Webpage | link |
| Name of the company | text |
| Facility in a commercial chain | YES / NO |
| City | text |
| Address | text |
| Phone number | text |
| E-mail | text |
| **Social media** | |
| Review in Google Maps | YES / NO |
| Rating in Google Maps | number between 1 to 5 |
| Facebook page | link |
| Facebook likes | number |
| Facebook followers | number |
| Instagram profile | link |
| Instagram followers | number |
| Instagram posts | number |
| Twitter page | link |
| Twitter followers | number |
| **Feature of the services described on the website** | |
| Information on years of experience | YES / NO |
| Experience | number |
| Information on course of the initial visit | YES / NO |
| Information on lab investigations performed before | YES / NO |
| Services are performed in own permise | YES / NO |
| Services are performed at the place indicated by the customer | YES / NO |
| Citations of scientific evidences on parenteral supplementation | YES / NO |

B)

| **Staff** | |
| --- | --- |
| Information about the personnel who perform the procedure | YES / NO |
| Among staff:   - Physician - Nurse and/or midwife - Dietetician - Cosmetologist - Paramedic - Physiotherapist - Psychologist | - YES / NO - YES / NO - YES / NO - YES / NO - YES / NO - YES / NO - YES / NO |
| **Indications for parenteral supplementation described on the website** | |
| - Vitamin and/or micronutrients deficiency - Physically active individuals - Fatigue - Excessive stress - Concentration loss or need of mental boost - Symptoms of depressive disorders - Hangover - Immunity enhancement - Dehydratation - Detoxification - Anti-aging - Beauty improvement - Weight loss - Improvement of libido - For specific diseases - Malignancy - Problems with fertility - Others | - YES / NO - YES / NO - YES / NO - YES / NO - YES / NO - YES / NO - YES / NO - YES / NO - YES / NO - YES / NO - YES / NO - YES / NO - YES / NO - YES / NO - YES / NO - YES / NO - YES / NO - text |

C)

| **Contraindications to parenteral supplementation described on the website** | |
| --- | --- |
| Information on contraindications | YES / NO |
| - Pregnancy - Breastfeeding - Hypersensitiveness - Certain drugs - Renal insufficiency - High blood pressure - Liver cirrhosis - Renal stones - Heart failure - Malignancy - Autoimmunologic diseases - Coagulation disorders - Being intoxicated - Epilepsy - Psychiatric disorders - Others | - YES / NO - YES / NO - YES / NO - YES / NO - YES / NO - YES / NO - YES / NO - YES / NO - YES / NO - YES / NO - YES / NO - YES / NO - YES / NO - YES / NO - YES / NO - text |

D)

| **Types of intravenous supplements** |  |
| --- | --- |
| Multi-ingredient infusions (cocktails) available | YES / NO |
| No information on exact cocktails ingredients | YES / NO |
| **Ingredients: electrolyts, micronutrients** |  |
| - Multi-electrolyte fluid - NaCl 0,9% - Magnesium - Selenium - Zinc - Iod - Chromium - Molibden - Iron - Copper - Calcium - Mangan | - YES / NO - YES / NO - YES / NO - YES / NO - YES / NO - YES / NO - YES / NO - YES / NO - YES / NO - YES / NO - YES / NO - YES / NO |
| **Ingredients: vitamins** |  |
| - A - B1 - B2 - B3 / PP - B5 - B6 - B7 - B9 / folic acid - B12 - C - D - E - K | - YES / NO - YES / NO - YES / NO - YES / NO - YES / NO - YES / NO - YES / NO - YES / NO - YES / NO - YES / NO - YES / NO - YES / NO - YES / NO |
| **Ingredients: others** | |
| - Alphalipoic acid - Gluthatione - Solcoseryl - Glucose - Aminoacids - Coenzym Q10 - Acetylocysteine - Choline - Sterile water - Antioxidants - Medications - Others | - YES / NO - YES / NO - YES / NO - YES / NO - YES / NO - YES / NO - YES / NO - YES / NO - YES / NO - YES / NO - YES / NO - text |
| Subcutaneouus supplements | text |
| Other types of services | text |
