# Supplementary material for: Characteristics of Websites Presenting Parenteral Supplementation Services in Five European Countries: A Cross-Sectional Study
Source: Nutrients. 2020 Nov 25;12(12):3614. doi: 10.3390/nu12123614 (PMC7760744; doi:10.3390/nu12123614)
Supplement: Supplementary file 1 [file nutrients-12-03614-s001.zip › Table S2.doc]

Table S2

Indications, contraindications, and ingredients mentioned in less than in 5% of websites in each country. Data presented as number (%).

| **Variables** | **All**  **n = 317** | **Czechia**  **n = 24** | **Ireland**  **n = 14** | **Italy**  **n = 58** | **Poland**  **n = 121** | **United Kingdom**  **n = 154** |
| --- | --- | --- | --- | --- | --- | --- |
| **Indications** | | | | | | |
| Problems with fertility [n] | 8 (2.2%) | 0 (0.0%) | 0 (0.0%) | 0 (0.0%) | 5 (4.1%) | 3 (2.0%) |
| **Contraindication for parenteral supplementation** | | | | | | |
| Autoimmunologic diseases [n] | 6 (1.6%) | 0 (0.0%) | 0 (0.0%) | 1 (1.7%) | 1 (0.8%) | 4 (2.6%) |
| Diabetes [n] | 5 (1.4%) | 0 (0.0%) | 0 (0.0%) | 0 (0.0%) | 2 (1.7%) | 3 (2.0%) |
| Breastfeeding [n] | 4 (1.1%) | 0 (0.0%) | 0 (0.0%) | 0 (0.0%) | 2 (1.7%) | 2 (1.3%) |
| Liver cirrhosis [n] | 4 (1.1%) | 0 (0.0%) | 0 (0.0%) | 0 (0.0%) | 3 (2.5%) | 1 (0.7%) |
| Active viral infection [n] | 4 (1.1%) | 0 (0.0%) | 0 (0.0%) | 2 (3.5%) | 2 (1.7%) | 0 (0.0%) |
| Malignancy [n] | 4 (1.1%) | 0 (0.0%) | 0 (0.0%) | 0 (0.0%) | 1 (0.8%) | 3 (2.0%) |
| Chronic illness [n] | 4 (1.1%) | 0 (0.0%) | 0 (0.0%) | 0 (0.0%) | 1 (0.8%) | 3 (2.0%) |
| High blood pressure [n] | 3 (0.8%) | 0 (0.0%) | 0 (0.0%) | 0 (0.0%) | 3 (2.5%) | 0 (0.0%) |
| Anaemia [n] | 2 (0.5%) | 0 (0.0%) | 0 (0.0%) | 0 (0.0%) | 2 (1.7%) | 0 (0.0%) |
| Heart damage [n] | 2 (0.5%) | 0 (0.0%) | 0 (0.0%) | 0 (0.0%) | 1 (0.8%) | 1 (0.7%) |
| Heart failure [n] | 2 (0.5%) | 0 (0.0%) | 0 (0.0%) | 0 (0.0%) | 2 (1.7%) | 0 (0.0%) |
| Epilepsy [n] | 2 (0.5%) | 0 (0.0%) | 0 (0.0%) | 0 (0.0%) | 1 (0.8%) | 1 (0.7%) |
| Coagulation disorders [n] | 1 (0.3%) | 0 (0.0%) | 0 (0.0%) | 0 (0.0%) | 1 (0.8%) | 0 (0.0%) |
| Low blood pressure [n] | 1 (0.3%) | 0 (0.0%) | 0 (0.0%) | 0 (0.0%) | 1 (0.8%) | 0 (0.0%) |
| Being intoxicated [n] | 1 (0.3%) | 0 (0.0%) | 0 (0.0%) | 0 (0.0%) | 1 (0.8%) | 0 (0.0%) |
| Psychiatric disorders [n] | 1 (0.3%) | 0 (0.0%) | 0 (0.0%) | 0 (0.0%) | 1 (0.8%) | 0 (0.0%) |
| Phenyloketonuria [n] | 1 (0.3%) | 0 (0.0%) | 0 (0.0%) | 0 (0.0%) | 1 (0.8%) | 0 (0.0%) |
| **Types of intravenous supplements** | | | | | | |
| Curcumin [n] | 5 (1.4%) | 0 (0.0%) | 0 (0.0%) | 0 (0.0%) | 2 (1.7%) | 3 (2.0%) |
| Sodium bicarbonate [n] | 3 (0.8%) | 0 (0.0%) | 0 (0.0%) | 0 (0.0%) | 1 (0.8%) | 2 (1.3%) |
| Arnica [n] | 3 (0.8%) | 0 (0.0%) | 0 (0.0%) | 0 (0.0%) | 3 (2.5% | 0 (0.0%) |
| Discus [n] | 3 (0.8%) | 0 (0.0%) | 0 (0.0%) | 0 (0.0%) | 3 (2.5%) | 0 (0.0%) |
| Chlor [n] | 2 (0.5%) | 0 (0.0%) | 0 (0.0%) | 0 (0.0%) | 2 (1.7%) | 0 (0.0%) |
| Sterile water [n] | 2 (0.5%) | 0 (0.0%) | 0 (0.0%) | 1 (1.7%) | 1 (0.8%) | 0 (0.0%) |
| Antioxidants [n] | 2 (0.5%) | 0 (0.0%) | 0 (0.0%) | 1 (1.7%) | 1 (0.8%) | 0 (0.0%) |
| Phosphorus [n] | 1 (0.3%) | 0 (0.0%) | 0 (0.0%) | 0 (0.0%) | 1 (0.8%) | 0 (0.0%) |
| Silicon [n] | 1 (0.3%) | 0 (0.0%) | 0 (0.0%) | 0 (0.0%) | 1 (0.8%) | 0 (0.0%) |
| Oliphenolia [n] | 1 (0.3%) | 0 (0.0%) | 0 (0.0%) | 0 (0.0%) | 1 (0.8%) | 0 (0.0%) |
| Amygdalin [n] | 1 (0.3%) | 0 (0.0%) | 0 (0.0%) | 0 (0.0%) | 1 (0.8%) | 0 (0.0%) |
