# Supplementary material for: Characteristics of Websites Presenting Parenteral Supplementation Services in Five European Countries: A Cross-Sectional Study
Source: Nutrients. 2020 Nov 25;12(12):3614. doi: 10.3390/nu12123614 (PMC7760744; doi:10.3390/nu12123614)
Supplement: Supplementary file 1 [file nutrients-12-03614-s001.zip › Table S3.doc]

Table S3

The other services offered in the analyzed facilities

| **Variables** | **All**  **n = 317** | **Czechia**  **n = 24** | **Ireland**  **n = 14** | **Italy**  **n = 58** | **Poland**  **n = 121** | **United Kingdom**  **n = 154** |
| --- | --- | --- | --- | --- | --- | --- |
| Beuty salon [n] | 77 (20.8%) | 8 (33.3%) | 4 (28.6%) | 9 (15.5%) | 5 (4.1%) | 51 (33.1%) |
| Ozone therapy [n] | 31 (8.4%) | 0 (0.0%) | 1 (7.1%) | 9 (15.5%) | 14 (11.6%) | 7 (4.6%) |
| Chelation therapy [n] | 10 (2.7%) | 0 (0.0%) | 1 (7.1%) | 0 (0.0%) | 4 (3.3%) | 5 (3.3%) |
| Acupuncture / acupressure [n] | 17 (4.6%) | 0 (0.0%) | 1 (7.1%) | 6 (10.3%) | 2 (1.7%) | 8 (5.2%) |
| Alcoholism treatment [n] | 13 (3.5%) | 0 (0.0%) | 0 (0.0%) | 0 (0.0%) | 12 (9.9%) | 1 (0.7%) |
